# Electro-Mechanical Cardiac Remodeling in Metabolic Syndrome: Association Between Frontal QRS-T Angle and Subclinical Left Ventricular Dysfunction Assessed by Four-Dimensional Echocardiography

**DOI:** 10.3390/jcm15135298

**Published:** 2026-07-07

**Authors:** Zeynep Ulutas, Yakup Yigit, Mirac Karaagac, Mehmet Cansel

**Affiliations:** 1 Department of Cardiology, Faculty of Medicine, Inonu University, Malatya 44280, Turkey; zeynep.ulutas@inonu.edu.tr; 2 Department of Cardiology, Faculty of Medicine, Malatya Turgut Ozal University, Malatya 44210, Turkey; ygtykp@gmail.com; 3 Agri Training and Research Hospital, Agrı 04200, Turkey; mirac.karaagac44@gmail.com

**Keywords:** metabolic syndrome, frontal QRS–T angle, four-dimensional strain echocardiography, electro-mechanical coupling

## Abstract

**Background/Objectives**: Metabolic syndrome (MS) has been demonstrated to be associated with subclinical myocardial dysfunction, which develops in the early stages. It has been hypothesised that traditional echocardiographic parameters may be insufficient in detecting these changes. The frontal QRS-T angle is an electrocardiographic parameter that is easily obtained and that reflects ventricular electrical heterogeneity. This study aimed to examine the relationship between the frontal QRS–T angle and left ventricular myocardial deformation, as assessed by four-dimensional strain echocardiography (4DSE), in patients with MS. **Methods**: The present cross-sectional study comprised 70 patients diagnosed with MS and 61 healthy individuals matched for age and gender. All participants underwent standard transthoracic and 4DSE examination and 12-lead electrocardiography. The values of global longitudinal strain (GLS), global circumferential strain (GCS), global radial strain (GRS) and global area strain (GAS) were analysed. The frontal QRS-T angle was calculated as the absolute difference between the QRS and T axes. The interrelationships between the variables were assessed using correlation and multivariate linear regression analyses. **Results**: Despite comparable left ventricular ejection fraction (LVEF) between the groups, 4DSE-derived myocardial deformation parameters, including GLS, GCS, GAS, and GRS, were significantly impaired in the MS group. The frontal QRS-T angle was found to be significantly higher in patients diagnosed with MS in comparison to the control group (*p* < 0.001). A significant correlation was identified between the frontal QRS-T angle and GLS (r = 0.498, *p* < 0.001). In multivariable linear regression analysis, the frontal QRS–T angle was independently associated with GLS (standardized β = 0.501, *p* < 0.001). In exploratory receiver operating characteristic (ROC) analysis, the frontal QRS–T angle showed moderate discriminatory ability for GLS-defined impaired myocardial deformation area under the curve (AUC) = 0.720). **Conclusions**: An increased frontal QRS–T angle in individuals with metabolic syndrome was independently associated with subclinical LV dysfunction despite preserved ejection fraction (EF). These findings suggest that the frontal QRS–T angle may serve as a simple and readily available electrocardiographic marker associated with impaired myocardial deformation, warranting further validation in larger prospective studies.

## 1. Introduction

Metabolic syndrome (MS) is a complex clinical condition characterised by the coexistence of interrelated cardiometabolic risk factors, such as abdominal obesity, insulin resistance, hypertension, and atherogenic dyslipidaemia [1]. MS represents a major global public health challenge, as it increases the risk of type 2 diabetes mellitus by approximately 5–7-fold, cardiovascular disease by nearly 3-fold, and all-cause mortality by about 1.5-fold [2]. Recent global meta-analyses have reported an adult MS prevalence ranging from 12% to 31%, depending on the diagnostic criteria used [3]. Between 2000 and 2023, the worldwide prevalence increased to 31.0% in women and 25.7% in men, corresponding to an estimated 1.54 billion affected adults [4].

Recent studies have demonstrated that MS is not confined to atherosclerotic processes, but is also associated with myocardial structural remodelling and functional impairment that commence prior to the development of clinically evident heart disease [5]. In particular, the following factors have been identified as contributing to myocardial fibrosis and early ventricular dysfunction: chronic low-grade inflammation, insulin resistance, increased oxidative stress and microvascular dysfunction [6,7].

The left ventricular ejection fraction (LVEF) is the most commonly used parameter in the assessment of systolic function, but it has limited sensitivity in detecting early myocardial damage [8]. In MS, subendocardial longitudinal fibres may be more sensitive to metabolic and inflammatory stress. This may lead to the development of subclinical systolic dysfunction despite a preserved ejection fraction [9]. In this context, myocardial deformation analysis, particularly global longitudinal strain (GLS), has emerged as a more sensitive method for detecting early left ventricular dysfunction and is recommended in current echocardiography guidelines [10,11]. The employment of sophisticated four-dimensional strain echocardiography (4DSE) techniques facilitates a comprehensive evaluation of myocardial mechanical function, thereby enabling a more detailed investigation of early cardiac remodelling [12,13]. Compared with conventional two-dimensional speckle-tracking echocardiography, 4DSE enables the assessment of multidirectional left ventricular deformation from a single volumetric dataset. By reducing geometric assumptions and the potential influence of through-plane motion, 4DSE may provide additional insight into subtle myocardial deformation abnormalities in patients with preserved LVEF, while complementing conventional echocardiographic and two-dimensional strain assessment [13].

Metabolic disorders are increasingly recognized to promote both mechanical and electrical myocardial remodeling. Myocardial fibrosis, alterations in intercellular coupling, and conduction heterogeneity that develop during the remodeling process contribute to abnormalities in ventricular depolarization and repolarization [14]. The frontal QRS-T angle is an electrocardiographic parameter that is easily obtained and that reflects the angular difference between ventricular depolarisation and repolarisation vectors. In recent years, there has been a resurgence of interest in this marker due to its association with elevated cardiovascular risk, arrhythmia, and mortality [15]. However, although previous studies have demonstrated associations between electrical abnormalities and impaired myocardial mechanics in overweight individuals, data specifically evaluating the relationship between the frontal QRS–T angle and 4DSE left ventricular deformation parameters in patients with MS remain limited. Therefore, the electromechanical relationship in this specific high-risk population has not been fully elucidated.

The objective of this study was to investigate the relationship between the frontal QRS–T angle and left ventricular myocardial deformation parameters, as assessed using 4DSE, in patients diagnosed with MS. We further hypothesized that the frontal QRS–T angle would be independently associated with impaired myocardial deformation despite preserved LVEF.

## 2. Methods

### 2.1. Study Design and Study Population

This was a single-centre, prospective, cross-sectional observational study conducted between October 2023 and January 2024. Consecutive adult individuals presenting to the cardiology outpatient clinic during the study period were screened for eligibility. Of the 172 individuals initially screened, 41 were excluded according to the predefined exclusion criteria. Of the 41 excluded individuals, 9 had known coronary artery disease, 13 had LVEF < 50%, 6 had atrial fibrillation or bundle branch block, 8 had inadequate echocardiographic image quality and 5 had chronic kidney disease. The final analytic sample included 131 participants, comprising 70 patients with metabolic syndrome and 61 control subjects. Patients fulfilling the National Cholesterol Education Program Adult Treatment Panel III (NCEP-ATP III)criteria for metabolic syndrome were enrolled in the MS group, whereas age- and sex-comparable individuals with normal clinical evaluation and without metabolic syndrome served as the control group. The study was approved by the İnönü University Faculty of Medicine Health Sciences Non-Interventional Clinical Research Ethics Committee under decision number 2023/58. Throughout the study, adherence to the principles of the Helsinki Declaration and the Good Clinical and Laboratory Practice guidelines was maintained.

The diagnosis of MS was made according to the NCEP-ATP III consensus criteria. In accordance with the established criteria, the following parameters were subjected to rigorous evaluation: serum triglyceride levels of ≥150 mg/dL, or the initiation of medical intervention due to hypertriglyceridaemia; high-density lipoprotein (HDL)cholesterol levels of <50 mg/dL in women or <40 mg/dL in men, or the administration of treatment for this condition; arterial blood pressure of ≥130/85 mmHg, or the utilisation of antihypertensive treatment; and fasting plasma glucose levels of ≥100 mg/dL, or the existence of antidiabetic treatment. Individuals who satisfied a minimum of three of these criteria were designated as having MS and were thus included in the study. The control group consisted of individuals who attended the cardiology outpatient clinic and had normal physical examination findings, electrocardiographic results, and echocardiographic evaluations. Eligible participants were recruited consecutively during the study period.

The study population comprised individuals aged 18 years and over. Individuals with known coronary artery disease, left ventricular ejection fraction < 50%, moderate or severe valve disease, cardiomyopathy, congenital heart disease, atrial fibrillation, bundle branch block or QRS duration ≥ 120 ms, permanent arrhythmia, chronic renal failure (estimated glomerular filtration rate [eGFR] < 30 mL/min/1.73 m^2^), active infection, or systemic inflammatory disease were excluded from the study.

### 2.2. Clinical and Laboratory Assessment

The demographic characteristics, anthropometric measurements, and blood pressure values of all participants were recorded in accordance with standard methods. Venous blood samples were collected after an eight-hour fast. Plasma glucose, creatinine, triglycerides, haemoglobin A1c(HbA1c), and complete blood count parameters were analysed in the hospital’s central biochemistry laboratory.

Standard 12-lead electrocardiogram (ECG) recordings were obtained from all participants. ECG recordings were performed with patients in a supine position after at least 10 min of rest, using the same device (Cardiofax V Model 9320, Nihon Kohden, Tokyo, Japan). The paper speed was set to 25 mm/s and the amplitude to 10 mm/mV. The evaluation of all recordings was conducted utilising the same ECG system, thereby ensuring the elimination of inter-device variability. The frontal plane QRS-T angle was calculated by taking the absolute value of the difference between the QRS axis and the T axis obtained from standard 12-lead ECG recordings. The determination of this parameter was achieved through the utilisation of axis values that were automatically reported by the ECG device. If the absolute difference between the QRS and T axes exceeded 180°, the frontal QRS–T angle was calculated as 360° minus the absolute difference. ECG tracings were reviewed for technical quality before analysis, and recordings containing ectopic beats or artifacts that could interfere with axis measurements were excluded. The automatically generated frontal QRS–T angle values were reviewed by an experienced cardiologist who was blinded to the echocardiographic findings.

### 2.3. Echocardiographic Assessment

Prior to conducting the echocardiographic assessment, it was imperative to ensure that all participants had undergone a sufficient period of rest. The transthoracic echocardiographic (TTE) assessment was then performed in the standard left lateral decubitus position. Imaging was performed using a commercially available ultrasound system (Vivid E95, GE Healthcare, Horten, Norway; Serial No: AU61302) in accordance with the recommendations of the American Society of Echocardiography (ASE). The examination was conducted utilising a phased array probe, which exhibited a frequency range of 1.4–4.6 MHz [8]. Appropriate image quality and frame rates were obtained from parasternal long- and short-axis views, as well as apical and subcostal windows. Imaging was conducted at the point of expiration and across three consecutive cardiac cycles. All participants were evaluated under the auspices of simultaneous electrocardiographic monitoring. The diameters of the left ventricle (LV) (diastolic and systolic), the left atrium, and the right heart were obtained from parasternal long-axis and apical views. Mitral inflow velocities were measured using the pulsed-wave Doppler method, and E and A wave velocities were recorded. Right ventricular systolic function was assessed by tricuspid annular plane systolic excursion (TAPSE). Systolic pulmonary artery pressure (SPAP) was calculated from the tricuspid regurgitation jet velocity.

Left ventricular mass was calculated using the formula: 0.8 × 1.04 × [(LVEDD + IVSd + PWTd)^3^ − LVEDD^3^] + 0.6 g, whereleft ventricular end-diastolic diameter (LVEDD), interventricular septal thickness in diastole (IVSd), and posterior wall thickness in diastole (PWTd)were measured in centimeters. Left ventricular mass index (LVMI)was obtained by indexing LV mass to body surface area. Body surface area was calculated using the Mosteller formula. Relative wall thickness was calculated as 2 × PWTd/LVEDD.

### 2.4. Four-Dimensional Strain Echocardiography (4DSE) Left Ventricular Assessment

4DSE data sets pertaining to the LV were obtained using the full-volume imaging technique with a matrix-array 4D probe through the apical window (Vivid E95, GE Healthcare, Horten, Norway; Serial No: AU61302). Image recordings were performed under simultaneous electrocardiographic monitoring, by combining 4–6 consecutive cardiac cycles acquired at the end of expiration with breath-hold (multibeat acquisition). Recordings were obtained at a rate of at least 25 images per second (≥25 frames/sec) to ensure optimal image quality and were transferred to a digital medium.

The 4DSE data sets that were acquired were evaluated offline using specialised analysis software (EchoPAC PC, Version 8, GE Healthcare). During the analysis, the LV endocardial borders were defined using a semi-automatic algorithm, with manual corrections made where necessary. Consequently, the analysis yielded the calculation of LV end-diastolic volume (EDV), end-systolic volume (ESV) and LVEF. Furthermore, global longitudinal strain (GLS), global circumferential strain (GCS), global radial strain (GRS), and global area strain (GAS) parameters were obtained as part of the myocardial deformation analysis. Left ventricular strain analysis was performed according to the current recommendations of the American Society of Echocardiography (ASE), and myocardial deformation parameters were interpreted in accordance with these guideline recommendations [11].

In order to evaluate the repeatability of echocardiographic and electrocardiographic measurements, analyses of intra-observer and inter-observer variability were performed. For intra-observer variability, measurements were repeated by the same observer in 20 randomly selected participants after a two-week interval while blinded to the initial results. For inter-observer variability, the same datasets were independently analyzed by a second experienced observer who was unaware of the first observer’s measurements. Intraclass correlation coefficients (ICCs) were calculated to assess reproducibility. Twenty participants were randomly selected using a computer-generated random list for reproducibility analysis. A high level of agreement was observed for GLS measurements (intraobserver ICC = 0.94, 95% CI: 0.86–0.98; interobserver ICC = 0.89, 95% CI: 0.75–0.95). For frontal QRS–T angle measurements, intraobserver ICC was 0.96 (95% CI: 0.90–0.98), and interobserver ICC was 0.93 (95% CI: 0.83–0.97).

### 2.5. Statistical Method

All statistical analyses were performed using SPSS software (version 22.0, IBM Corp., Armonk, NY, USA). Continuous variables were assessed for normal distribution using the Kolmogorov–Smirnov test. Normally distributed variables were expressed as mean ± standard deviation (SD), while categorical variables were presented as numbers and percentages. Comparisons between the MS and control groups were performed using the independent samples *t*-test for continuous variables. Categorical variables were compared using the chi-square test or Fisher’s exact test, as appropriate. Non-normally distributed continuous variables were compared using the Mann–Whitney U test and expressed as median and interquartile range. GLS was analyzed using signed values; therefore, higher, less negative GLS values indicate worse myocardial deformation. Correlations between frontal QRS–T angle and clinical, laboratory, and strain parameters were evaluated using Pearson correlation analysis. To determine independent predictors of GLS, variables showing statistical significance in univariate analysis were entered into a multivariable linear regression model using the enter method. Covariates included in the multivariable linear regression model were selected based on clinical relevance and their known association with metabolic syndrome and myocardial deformation, rather than solely on univariate statistical significance. Standardized beta (β) coefficients were calculated to assess the relative strength of associations. Subclinical left ventricular dysfunction was defined as impaired myocardial deformation despite preserved left ventricular ejection fraction. Impaired GLS was defined as a GLS value less negative than −18% (absolute GLS < 18%). Accordingly, participants with preserved LVEF and GLS > −18% were classified as having subclinical LV dysfunction for the ROC analysis. The discriminatory performance of the frontal QRS–T angle for GLS-defined impaired myocardial deformation was explored using receiver operating characteristic (ROC) curve analysis. The area under the curve (AUC) was calculated with 95% confidence intervals (CI), and the optimal cut-off value was determined using the Youden index. A two-tailed *p* value < 0.05 was considered statistically significant.

An a priori sample size calculation was performed using G*Power, version 3.1.9.7 (Heinrich-Heine-Universität Düsseldorf, Düsseldorf, Germany). Assuming a moderate correlation coefficient of r = 0.30 between the frontal QRS–T angle and GLS, with a two-sided alpha of 0.05 and 80% power, the minimum required sample size was calculated as 84 participants. Therefore, the final sample size of 131 participants was considered sufficient for the primary correlation analysis.

## 3. Results

A total of 131 individuals were included in the study; 70 of them had MS, while 61 were in the control group. No statistically significant differences were observed between the groups with respect to age and gender distribution (*p* > 0.05). However, a significant elevation in body mass index and systolic and diastolic blood pressure values was observed in the MS group compared to the control group (*p* < 0.001).

### 3.1. Clinical and Laboratory Findings

Compared with the control group, patients with metabolic syndrome had significantly higher fasting glucose, triglyceride, and HbA1c levels (all *p* < 0.001). Serum creatinine, white blood cell count, hemoglobin, and platelet levels were comparable between the groups (all *p* > 0.05) (Table 1).

### 3.2. Conventional Echocardiographic Findings

In conventional echocardiographic assessment, the anterior–posterior diameter of the left atrium was found to be significantly wider in the MS group (*p* < 0.001). In conventional echocardiographic assessment, LV diastolic and systolic diameters were slightly but significantly higher in the MS group. Left atrial diameter, LV mass index, and relative wall thickness were also significantly increased in patients with MS. Mitral inflow analysis demonstrated lower E-wave velocity and higher A-wave velocity in the MS group, whereas right atrial diameter, tricuspid flow velocity, SPAP, and TAPSE were comparable between the groups (Table 2).

### 3.3. Four-Dimensional Left Ventricular Function Analysis

**Figure 1:**
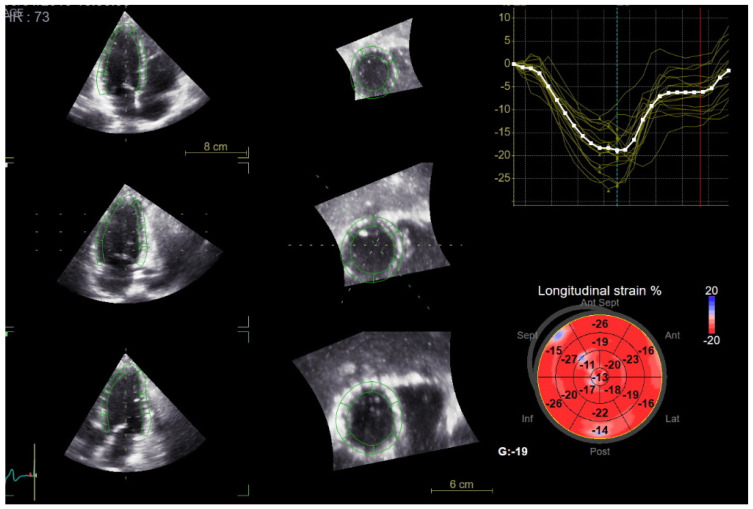
Representative example of four-dimensional speckle-tracking echocardiographic analysis of the left ventricle, demonstrating three-dimensional endocardial tracking and quantitative assessment of global strain parameters (GLS). The thin colored solid curves represent segmental longitudinal strain, whereas the thick white dashed curve represents the global longitudinal strain curve.

**Figure 2:**
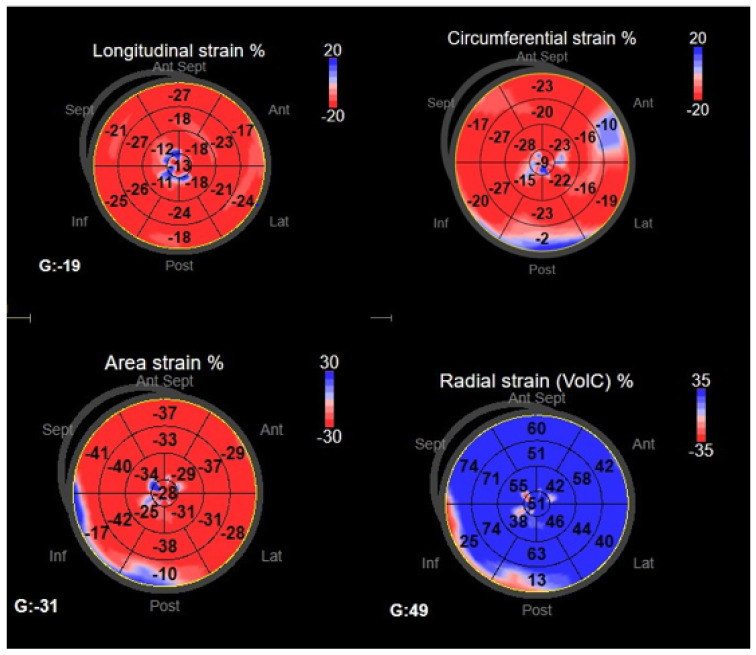
Representative four-dimensional speckle-tracking echocardiographic analysis of left ventricular myocardial deformation. The figure displays color-coded strain maps and quantitative measurements of global longitudinal strain (GLS), global circumferential strain (GCS), global radial strain (GRS), and global area strain (GAS).

In the four-dimensional echocardiography analysis, LV end-diastolic volume, LV end-systolic volume, and LVEF were comparable between the groups. A representative example of the 4DSE analysis is shown in Figure 1. Despite similar LV volumes and preserved ejection fraction, all 4DSE-derived myocardial deformation parameters showed significant impairment in the MS group. GLS, GCS, GAS, and GRS were significantly impaired in patients with MS compared with controls (Table 2; Figure 2).

### 3.4. Electrocardiographic Findings

Electrocardiographic evaluation revealed that the frontal QRS–T angle was significantly increased in patients with MS compared to the control group (48.1° ± 23.1° vs. 19.5° ± 11.5°, *p* < 0.001). No significant difference was observed between the groups in terms of QT and corrected QT (QTc) intervals (*p* > 0.05) (Table 2).

### 3.5. Correlation Analysis

A moderate positive correlation was identified in the correlation analysis between the frontal QRS-T angle and GLS (r = 0.498, *p* < 0.001). Because signed GLS values were used, this positive correlation indicates that a higher frontal QRS–T angle was associated with less negative, and therefore more impaired, GLS values. Furthermore, the frontal QRS-T angle was found to be positively associated with body mass index, systolic and diastolic blood pressure, fasting glucose, and triglyceride levels (all *p* < 0.05) (Table 3).

### 3.6. Regression Analysis

In univariate linear regression analysis, the frontal QRS–T angle, body mass index, triglycerides, glucose, systolic and diastolic blood pressure values were found to be significantly associated with GLS (*p* < 0.05). In multivariate linear regression analysis, the frontal QRS–T angle remained independently associated with GLS in the multivariable model (standardized β = 0.501, *p* < 0.001). The body mass index (BMI) demonstrated a persistent independent relationship (β = 0.263, *p* = 0.039). The triglyceride, glucose, and blood pressure variables lost their statistical significance in the multivariate model (see Table 4). Multicollinearity was assessed using tolerance and variance inflation factor (VIF) values, and regression assumptions were evaluated by residual plots and residual normality. Because of the modest sample size, robust standard errors and a sex-adjusted sensitivity model were additionally applied. No relevant multicollinearity was observed; VIF values ranged from 1.15 to 1.75, and tolerance values ranged from 0.570 to 0.873. The association between the frontal QRS–T angle and GLS remained statistically significant in the robust analysis.

After adding LVMI to the multivariable regression model, the frontal QRS–T angle remained independently associated with GLS (β = 0.042, standardized β = 0.550, *p* = 0.020). This association also remained statistically significant in the robust standard error sensitivity analysis. LVMI itself was not independently associated with GLS in this model.

### 3.7. ROC Curve Analysis

**Figure 3:**
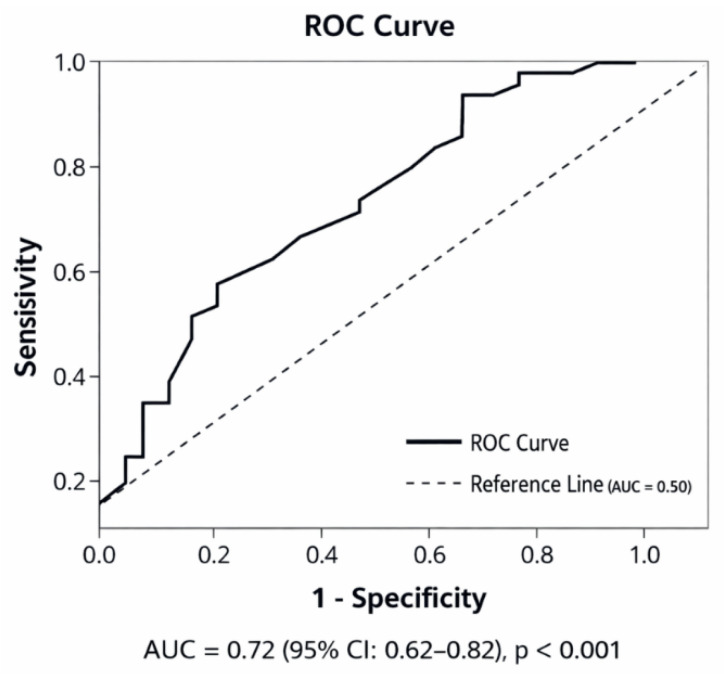
Receiver operating characteristic (ROC) curve illustrating the discriminatory ability of the frontal QRS–T angle for identifying impaired myocardial deformation in the study population.

Using this predefined GLS-based definition of subclinical left ventricular dysfunction, ROC curve analysis demonstrated moderate discriminatory ability of the frontal QRS–T angle for identifying impaired myocardial deformation (AUC = 0.720, 95% CI: 0.62–0.82, *p* < 0.001). The 36.5° threshold determined by the Youden index yielded 65.8% sensitivity and 66.7% specificity (Figure 3).

## 4. Discussion

In the present study, patients with MS exhibited significant impairment in 4DSE-derived myocardial deformation parameters, including GLS, GCS, GAS, and GRS, despite preserved LVEF. In addition, the frontal QRS–T angle was significantly wider in the MS group and showed a significant association with GLS. Multivariate analysis demonstrated that the frontal QRS–T angle was independently associated with impaired GLS in patients with MS. Collectively, these findings suggest that mechanical and electrical myocardial remodeling may coexist in patients with MS.

MS is recognised as a systemic cardiometabolic condition that affects myocardial structure and function in the early stages, not limited solely to atherosclerotic processes [16]. It has been demonstrated that metabolic inflammation, insulin resistance, and increased oxidative stress can disrupt myocardial energy metabolism, resulting in interstitial fibrosis and microvascular dysfunction [17]. As these pathophysiological processes primarily affect the subendocardial longitudinal myocardial fibres, longitudinal function may deteriorate in the early stages, even if the LVEF is preserved [11]. Accordingly, GLS is considered a more sensitive marker than LVEF for detecting early myocardial dysfunction in metabolic disorders [18].

In the present study, despite preserved LVEF in the MS group, significant impairment in GLS, GCS, GAS, and GRS values supports the presence of early myocardial mechanical dysfunction in MS. These findings are consistent with those of previous studies reporting the use of deformation imaging methods to detect subclinical cardiac dysfunction in MS [19]. 4DSE facilitates a three-dimensional (3D) spatial analysis of myocardial deformation, thereby enabling a more precise assessment of regional and global mechanical changes [20]. Consequently, 4DSE may provide incremental insight into early cardiac remodeling in MS. Increased inflammation, lipotoxicity, and interstitial fibrosis can lead to myocardial conduction heterogeneity, which in turn disrupts the coordination between depolarisation and repolarisation processes [14]. In addition, chronic low-grade inflammation, oxidative stress, and insulin resistance promote extracellular matrix remodeling and diffuse myocardial fibrosis, leading to impaired myocardial deformation and altered ventricular depolarization–repolarization patterns. This electromechanical remodeling may provide a plausible explanation for the observed association between an increased frontal QRS–T angle and impaired GLS in patients with MS [21].

The frontal QRS–T angle is a simple electrocardiographic marker that reflects the spatial difference between ventricular depolarization and repolarization vectors and is considered an indicator of electrical heterogeneity [22]. Previous large cohort studies have demonstrated that a widened frontal QRS–T angle is associated with ventricular arrhythmias, structural heart disease, and adverse cardiovascular outcomes [23,24]. These observations support the concept that electrical remodeling may occur in parallel with mechanical myocardial dysfunction, providing a potential electrophysiological correlate of impaired myocardial deformation [23,25].

Previous studies in overweight individuals have demonstrated that subclinical LV mechanical dysfunction can be detected by three-dimensional strain analysis despite preserved conventional echocardiographic parameters, and that these alterations are associated with a wider frontal QRS–T angle [26]. Our findings extend these observations to patients with MS by demonstrating a significant association between the frontal QRS–T angle and GLS. Taken together, these results support the concept that electrical and mechanical myocardial remodeling are closely interconnected rather than independent processes. Nevertheless, the frontal QRS–T angle should be regarded as a nonspecific marker of electrical heterogeneity, as it may be influenced by multiple structural and electrophysiological abnormalities, including ventricular hypertrophy, myocardial fibrosis, ischemia, and conduction disturbances. Therefore, its interpretation should always be made within the appropriate clinical context.

Our findings suggest that an increased frontal QRS–T angle may reflect underlying electromechanical remodeling associated with impaired myocardial deformation in patients with MS. Importantly, the association between the frontal QRS–T angle and GLS remained significant after adjustment for LVMI, suggesting that this relationship was not explained solely by increased left ventricular mass or geometric remodeling. Given the single-center and cross-sectional design of the present study, these findings should be interpreted as exploratory and hypothesis-generating. Although the frontal QRS–T angle was associated with impaired myocardial deformation, the present results do not establish causality and do not support its use as a stand-alone screening or diagnostic tool. Rather, the frontal QRS–T angle may provide complementary information regarding subclinical electromechanical remodeling in patients with MS. The ROC-derived cut-off value of 36.5° should therefore be interpreted as a study-specific threshold for GLS-defined impaired myocardial deformation rather than as a universal abnormal QRS–T angle cut-off. Further prospective, multicenter studies are required to determine whether this readily obtainable electrocardiographic parameter provides incremental clinical value beyond conventional clinical and echocardiographic assessment [27].

Multivariable analysis demonstrated that body mass index remained independently associated with GLS, highlighting the potential contribution of obesity to early myocardial remodeling. Inflammation, lipotoxicity, and increased hemodynamic load associated with excess adipose tissue may contribute to subclinical myocardial dysfunction independently of other components of MS. Conversely, the loss of statistical significance of triglycerides, glucose, and blood pressure variables in the multivariable model may indicate that their effects on myocardial function are largely mediated through shared metabolic pathways related to obesity rather than acting as independent determinants.

The present study has several limitations. First, its single-center, cross-sectional design precludes causal inference and does not allow evaluation of the prognostic significance of the observed association between the frontal QRS–T angle and myocardial deformation parameters. Second, advanced tissue characterization techniques, such as cardiac magnetic resonance imaging, were not available; therefore, the structural mechanisms underlying electrical and mechanical remodeling could not be directly assessed. In addition, the relatively modest sample size and single-center recruitment may limit the generalizability of our findings and introduce the possibility of selection bias. Although multivariable analyses were performed, residual confounding by unmeasured variables cannot be completely excluded. Another important limitation is the lack of detailed information regarding concomitant antihypertensive, lipid-lowering, and antidiabetic therapies. These medications may influence myocardial deformation through effects on loading conditions, metabolic control, vascular function, inflammation, and myocardial remodeling. In addition, some therapies may affect electrocardiographic indices, including ventricular repolarization-related parameters. Therefore, although the present study demonstrated an association between the frontal QRS–T angle and impaired myocardial deformation, the potential confounding effect of background medical therapy could not be fully evaluated and should be considered when interpreting the findings. Furthermore, findings derived from 4DSE analysis may not be directly generalizable to other imaging platforms because of methodological and software-related differences. Moreover, image quality and acoustic window limitations, particularly in individuals with higher body mass index, may influence tracking performance and represent an inherent technical limitation of 4DSE. Larger prospective multicenter studies incorporating advanced tissue characterization techniques and external validation cohorts are warranted to validate these findings and further elucidate the relationship between electrical and mechanical remodeling in metabolic syndrome.

In conclusion, patients with MS exhibited impaired myocardial deformation despite preserved LVEF, indicating the presence of subclinical myocardial dysfunction. The significant association between the frontal QRS–T angle and GLS suggests that this simple and readily obtainable electrocardiographic parameter may reflect underlying electromechanical remodeling and may provide complementary information regarding subclinical electromechanical remodeling in patients with MS. However, these findings should be considered exploratory and require validation in larger prospective multicenter studies before clinical interpretation.

## Figures and Tables

**Table 1:** Baseline Demographic, Clinical, and Laboratory Characteristics of the Study Population.

Variable	Control (*n* = 61)	Patient (*n* = 70)	*p* Value
**Demographic and Clinical Variables**			
Age (years)	46.67 ± 9.65	47.88 ± 11.99	0.546
Body mass index (kg/m^2^)	24.46 ± 2.90	30.47 ± 4.31	<0.001
Male sex, *n* (%)	25 (41.0%)	22 (31.4%)	0.340
Systolic BP (mmHg)	114.50 ± 10.58	130.45 ± 13.61	<0.001
Diastolic BP (mmHg)	71.82 ± 7.65	81.00 ± 10.13	<0.001
**Laboratory Parameters**			
Glucose (mg/dL)	90.60 ± 13.94	109.37 ± 28.60	<0.001
Creatinine (mg/dL)	0.83 ± 0.16	0.83 ± 0.15	0.975
Triglyceride (mg/dL)	99.44 ± 46.17	161.19 ± 69.14	<0.001
HDL cholesterol (mg/dL)	49.86 ± 8.25	47.77 ± 8.89	0.220
HbA1c (%)	5.61 ± 0.39	6.59 ± 1.17	<0.001
WBC (10^3^/µL)	7.16 ± 1.57	7.25 ± 1.29	0.750
Hemoglobin (g/dL)	13.55 ± 1.42	13.38 ± 1.42	0.548
Platelet (10^3^/µL)	244.96 ± 65.83	250.50 ± 60.38	0.655

**Table 2:** Comparison of Echocardiographic and Electrocardiographic Parameters Between the Control and Patient Groups.

Variable	Control (*n* = 61)	Patient (*n* = 70)	*p* Value
**Conventional Echocardiography**			
LVDD (mm)	46.5 ± 3.4	47.8 ± 3.5	0.034
LVSD (mm)	29.2 ± 4.1	30.7 ± 3.8	0.032
LV mass index, g/m^2^	88.16 ± 22.01	101.53 ± 21.77	<0.001
Relative wall thickness	0.44 ± 0.10	0.51 ± 0.12	<0.001
LA diameter (mm)	34.2 ± 3.7	37.1 ± 2.8	<0.001
Mitral E (cm/s)	83.3 ± 17.8	70.0 ± 15.1	<0.001
Mitral A (cm/s)	61.3 ± 12.7	70.5 ± 13.8	<0.001
Right atrial diameter (mm)	36.4 ± 4.1	35.9 ± 4.6	0.515
Tricuspid velocity (cm/s)	218.6 ± 21.7	213.0 ± 25.6	0.183
SPAP (mmHg)	22.9 ± 3.8	23.2 ± 3.9	0.657
TAPSE (mm)	22.9 ± 2.3	22.3 ± 3.8	0.285
4DSE Left Ventricular Parameters			
EDV (mL)	115.1 ± 24.3	118.8 ± 28.0	0.424
ESV (mL)	45.7 ± 13.4	46.4 ± 14.0	0.771
LVEF (%)	61.2 ± 3.5	61.0 ± 3.8	0.756
GLS (%)	−19.9 ± 2.0	−16.8 ± 1.4	<0.001
GCS (%)	−20.1 ± 3.3	−18.1 ± 2.9	<0.001
GAS (%)	−38.1 ± 4.5	−32.9 ± 5.4	<0.001
GRS (%)	43.7 ± 8.4	40.3 ± 6.4	0.010
**Electrocardiographic Parameters**			
QRS–T angle (°)	19.5 ± 11.5	48.1 ± 23.1	<0.001
QT (ms)	363.3 ± 28.9	358.8 ± 22.5	0.319
QTc (ms)	399.1 ± 15.9	397.8 ± 24.2	0.721

**Table 3:** Correlation Analysis Between the Frontal QRS–T Angle, Global Longitudinal Strain, and Clinical Variables.

Variable	r	*p* Value
Global longitudinal strain (GLS)	0.498	<0.001
Body mass index (kg/m^2^)	0.412	<0.001
Systolic blood pressure (mmHg)	0.381	<0.001
Diastolic blood pressure (mmHg)	0.280	0.005
Glucose (mg/dL)	0.314	0.001
Triglyceride (mg/dL)	0.225	0.023

**Table 4:** Univariate and Multivariable Linear Regression Analyses for Determinants of Global Longitudinal Strain.

Variable	Univariate β (95% CI)	*p* Value	Multivariable β (95% CI)	Standardized β	*p* Value
Frontal QRS–T angle (°)	0.042 (0.024–0.060)	<0.001	0.038 (0.020–0.056)	0.501	<0.001
Body mass index (kg/m^2^)	0.135 (0.039–0.231)	0.008	0.112 (0.010–0.214)	0.263	0.039
Triglyceride (mg/dL)	0.010 (0.002–0.018)	0.019	0.007 (−0.000–0.014)	0.224	0.064
Glucose (mg/dL)	0.023 (0.003–0.043)	0.024	0.015 (−0.005–0.035)	0.198	0.159
Systolic BP (mmHg)	0.044 (0.008–0.080)	0.020	0.024 (−0.017–0.065)	0.160	0.269
Diastolic BP (mmHg)	0.069 (0.015–0.123)	0.015	0.004 (−0.051–0.059)	0.020	0.881

Model performance: R^2^ = 0.633, adjusted R^2^ = 0.615, *p* < 0.001. Regression assumptions were assessed using residual plots and residual normality.

## Data Availability

The datasets generated and/or analyzed during the current study are available from the corresponding author on reasonable request.
